# Work-focused cognitive behavioral intervention for psychological complaints in patients on sick leave due to work-related stress: Results from a randomized controlled trial

**DOI:** 10.1186/s12952-017-0078-z

**Published:** 2017-08-22

**Authors:** Vita Ligaya Dalgaard, Lars Peter Sønderbo Andersen, Johan Hviid Andersen, Morten Vejs Willert, Ole Carstensen, David John Glasscock

**Affiliations:** 1 Danish Ramazzini Centre, Department of Occupational Medicine, The Regional Hospital West Jutland - University Research Clinic, Herning, Denmark; 2 Danish Ramazzini Centre, Department of Occupational Medicine, Aarhus University Hospital, Aarhus, Denmark

**Keywords:** Perceived stress, Cognitive behavioral therapy, Absenteeism, Randomized controlled trial, Mental health, Sleep, Cognitive difficulties

## Abstract

**Background:**

Work-related stress is a global problem with negative implications for individuals and society. The purpose of the current study was to evaluate a stress management intervention for patients on sick leave due to work-related stress complaints using a three-armed randomized controlled design.

**Methods:**

Participants were patients referred from three municipalities to the regional Department of Occupational Medicine. Inclusion criteria were: 1) sick leave due to work-related stress complaints, 2) a diagnosis of adjustment disorder or reactions to severe stress (ICD 10 code: F43,2 – F 43,9 not PTSD) or mild depressive episode (F 32.0). Through a double randomization procedure patients (*n* = 163) were randomized to either an intervention group (*n* = 58), a ‘control group A’ receiving a clinical examination (*n* = 56), or ‘control group B’ (*n* = 49) receiving no offers at the department. The intervention comprised six sessions of individual cognitive behavioral therapy and the offer of a small workplace intervention. Questionnaire data were analyzed with multivariate repeated measurements analysis. Primary outcomes assessed were perceived stress and general mental health. Secondary outcomes were sleep quality and cognitive failures. Follow-up was at four and 10 months after baseline.

**Results:**

Complaints were significantly reduced in all groups over time. No group effects were observed between the intervention group and control group A that was clinically assessed. Significant group effects were found for perceived stress and memory when comparing the intervention group to group B, but most likely not due to an intervention effect.

**Conclusion:**

Psychological complaints improved substantially over time in all groups, but there was no significant treatment effect on any outcomes when the intervention group was compared to control group A that received a clinical assessment.

**Trial registration:**

ISRCTN ISRCTN91404229. Registered 03 August 2012 (retrospectively registered).

**Electronic supplementary material:**

The online version of this article (doi:10.1186/s12952-017-0078-z) contains supplementary material, which is available to authorized users.

## Background

Work-related stress is pervasive in modern work life with many negative individual and societal implications [1, 2]. Workplaces have changed extensively during the last decades due to globalisation, new technologies and increased performance requirements [3–5]. European countries have seen increasing numbers of disability pensions and sick leave due to mental health problems [6]. Similarly, Danish departments of occupational medicine have observed a rise in patients with work-related stress complaints. Patients are typically on sick leave and many are diagnosed with adjustment disorder, exhibiting stress symptoms associated with impaired functioning at work and at home [7]. It is imperative that effective treatment options are available and thus many researchers and clinicians have engaged in this area during the last decade [2].

“Stress” has several connotations but is commonly defined as the experience of demands or pressures exceeding individual coping resources, thereby threatening personal wellbeing [8, 9]. Work-related stress refers to the experience of demands and pressures related to work, e.g. high work load or interpersonal problems. Ongoing stress involves affective, cognitive, physiological, and behavioral changes with the risk of impaired functioning and reduced work capability [10, 11].

Meta-analyses have found cognitive behavioral therapy (CBT) superior in reducing stress levels and psychological complaints among stressed workers compared to other types of intervention [1, 12, 13]. However, most studies have included volunteers and samples that were not on sick leave [1, 12]. It remains unclear whether these findings apply to clinical samples with work-related stress complaints [1, 12]. A few randomized controlled trials (RCT) have included participants on sick leave due to work stress. These indicate that psychological complaints improve over time, especially during the first few months of recovery, but with regard to intervention effects, the studies show mixed results [14–20]. For example, van der Klink et al. [14] employed a randomized cluster design but did not find a CBT-based activating intervention superior to care-as-usual in reducing psychological symptoms. Using a three armed RCT design, Blonk et al. [15] compared extensive CBT to a brief CBT-based intervention (aimed at both the individual and the workplace) and a third (no-treatment) group that received only two sessions with a general practitioner, but found no psychological outcome differences between the groups. Similar results were reported in an RCT by de Vente et al. [16], where group-based CBT, individual CBT and care-as-usual were evaluated with regard to symptom improvement and length of sick leave. Furthermore, using the same intervention manual as in the current study, Dalgaard et al. [17] reported non-significant borderline effects on sleep and cognitive complaints in stressed individuals referred from their general practitioner when compared to treatment as usual. More positive results have been achieved in wait-list controlled trials. One study found group-based CBT more effective in reducing perceived stress, sleep complaints and cognitive failures than a wait list condition [18, 19]. In another study by Netterstrøm et al. [20], individual CBT combined with mindfulness and workplace dialogue was more effective in reducing psychological complaints compared to a wait-list group but not when compared to treatment as usual. The study by Netterstrøm et al. [20] allowed for inclusion of participants with major depression and may therefore be less comparable to the current study. In summary, psychological complaints seem to improve over time after a stress-related sick leave notification; either through natural progression or possibly by treatment.

The aim of the current study was to evaluate the effectiveness of a stress management intervention in reducing psychological complaints (perceived stress, general mental health, cognitive failures and sleep problems) among employees on sick leave due to work-related stress. The intervention comprised individual work-focused CBT in combination with the offer of a small workplace intervention/meeting. While a thorough clinical examination was employed to ensure that participants fulfilled inclusion criteria, the assessment procedure, which involved discussing the patient’s work situation, experiences of stress and coping, might in itself represent a mini-intervention. For this reason two control groups (one receiving baseline clinical assessment only and one receiving neither assessment nor treatment) were employed. We hypothesized that the intervention would be superior to the two control groups in reducing psychological stress complaints.

## Methods

### Procedure

**Fig. 1:**
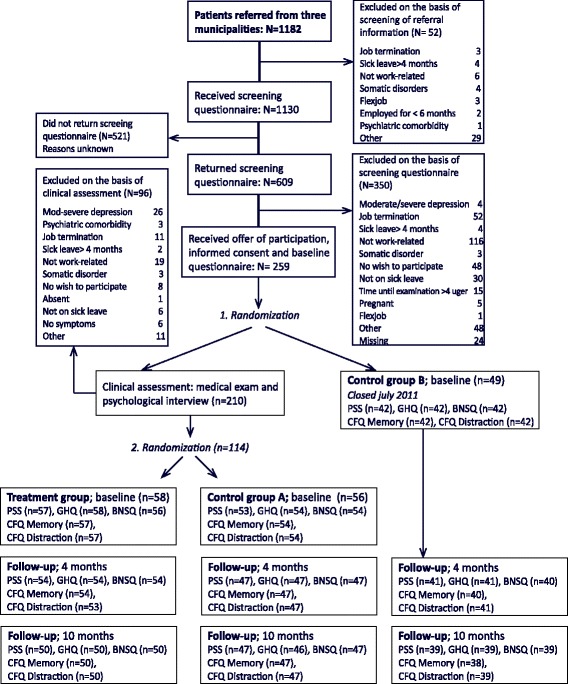
Flowchart of patient recruitment, allocation and outcome assessment. Trial period: 1st June 2009– 31st of Februrary 2014. Numbers at follow-up refer to those who responded to follow-up questionnaires and were analyzed

The study was designed as a prospective randomized controlled trial with three groups, a treatment group and two control groups. The 163 participants were randomized into either the intervention group (*n* = 58), control group A, who received clinical assessment but no treatment (*n* = 56), or control group B (*n* = 49), who received no offers at the department. Additional information on recruitment, allocation, and outcome assessments is outlined in Fig. 1.

### Participants

Patients were referred by the sickness benefits departments of three local municipalities to a regional Department of Occupational Medicine in Denmark. Potential participants (*N* = 1182) were referred to the department by a contact person from each of the three municipalities, but 52 individuals were excluded on the basis of the written referrals (see Fig. 1 for reasons). The remainder received a screening questionnaire along with information about the project. The questionnaire addressed employment, health status and whether respondents’ symptoms, in their opinion, were related to stressors at work. Questionnaires were returned by mail. Participants fulfilling inclusion criteria received a letter with informed consent, an offer to participate and a baseline questionnaire. The letter included instructions to mail back the consent form and baseline questionnaire. Subsequently, the first randomization was conducted, and participants were randomly assigned either to clinical assessment or control group B (see Figure 1). Thus, all patients filled in baseline questionnaires before the randomization. However, during the inclusion period it became clear that far more participants than anticipated were excluded as a result of the clinical assessment (e.g. their stress condition was not sufficiently work-related). This raised the possibility that the two control groups would not be comparable, since a similar proportion of participants in control group B would presumably have been excluded, had they also been thoroughly assessed. We therefore stopped further selection into control group B in July 2011, when it contained 49 participants. This meant that all new potential participants were invited to the clinical assessment on the basis of the screening questionnaire. Thus, all subsequent patients were randomized after clinical assessment if included in the study. Even though control group B is potentially different from the intervention group and control group A, we have chosen to report the results from all groups in accordance with the original study design, while recognizing potential limitations in group B.

The study design originally involved two randomization procedures. After receiving the screening questionnaire, the project secretary gave each potential participant a number between 1 and 99,999. Numbers were generated in True Random Number Generator (www.random.org) (Participants randomized to the clinical examination group: numbers 0–66,666 and control group B: numbers 66,667–99,999, see flow chart). Participants assigned to the clinical assessment group were invited to the clinic to determine further eligibility and subsequently randomized into either the treatment group or control group A. These participants received a number from a list of 1000 randomly generated numbers between 0 and 100.000. Group assignment was based on the sum of the digits of this number (the intervention group: unequal numbers and control group A: equal numbers). After randomization, participants in control groups A and B were followed with questionnaires only. Participants in both control groups were free to seek treatment elsewhere.

#### Inclusion and exclusion criteria

Patients who underwent the clinical assessment were included if they fulfilled the following criteria: (1) A diagnosis of adjustment disorder or reactions to stress (ICD 10 code: F43,2 – F 43,9, but not PTSD) or mild depression (F32.0) [7]. (2) Were on sick leave due to the above mentioned criteria. (3) The condition was evaluated by the psychologist as primarily work-related. (4) Patients planned on returning to their workplace. Exclusion criteria in the study were: (1) Co-morbidity of another psychiatric illness (e.g. moderate to severe depression). (2) Co-morbidity of a recently diagnosed chronic somatic disease. (3) Pregnancy. (4) Substance abuse. (5) Sick leave for more than 4 months prior to baseline. (6) Any degree of disability pension. (7) Job termination prior to baseline. (8) Employment <6 months. (9) Patients with major difficulties related to their private life were also excluded.

### The clinical assessment

The clinical assessment comprised a brief medical evaluation followed by a psychological interview. Medical examination was carried out by an occupational physician (OP) to rule out possible somatic causes of symptoms (e.g., diabetes, heart disease or thyroid condition). The examination followed a manual and lasted 15 to 30 min. Afterwards, the patient was seen by a trained psychologist who conducted a manualized interview lasting between 1 to 2 h. The interview covered work history; present work situation, work stressors; the current situation related to sick leave and symptom development. Non-work stressors, home life and dispositions for psychiatric illness were also addressed. The interview aimed to determine diagnosis and the likelihood of work-related stress having played a primary role in symptom development. Patients were not excluded if stressors related to non-work conditions were present, but they had to be of secondary nature.

### Intervention

The intervention consisted of an individual work-focused CBT program and an optional intervention/meeting at the workplace. The individual intervention consisted of six one hour sessions with a psychologist during a period of 16 weeks. The intervention was intended to strengthen the patient’s ability to cope with stressors at work. This involved (1) identifying work-related stressors, (2) modifying cognitions and behaviors related to the development of stress symptoms, (3) psycho-education about work-related stress, (4) homework assignments between each session. Treatment was carried out according to a manual, but the psychologist had some freedom in choosing among different techniques and homework assignments according to the clinical evaluation of the patient.

The workplace intervention comprised one or two meetings at the workplace with the patient, the psychologist, a leader and/or other representatives. The meeting took place during the treatment period and was intended to address stress-related problems at work and facilitate a process that would meet the needs of the patient when he/she returned to work. When the psychologist did not participate, the patient was helped in preparing to meet with the workplace. However, only six people in the intervention group accepted the offer of a direct workplace intervention. Therefore the intervention mainly consisted of work-focused individual CBT (Please see Dalgaard et al. [17] for a more detailed description of the intervention).

### Outcome measures

Outcomes were evaluated by self-report measures at baseline; after 4 months (corresponding to the completion of the intervention), and at 10 months after baseline. Non-responders were given two reminders. Primary outcome measures were the Perceived Stress Scale (PSS-10) [21] and the General Health Questionnaire (GHQ-30) [22].

The PSS-10 is a validated measure of global stress [21]. The scale consists of 10 items and measures the degree to which participants assess life as unpredictable, uncontrollable, and overwhelming. Items are rated on a 5-point Likert scale ranging from 0 (never) to 4 (very often) (total range: 0–40). Patients answered according to their experience during the previous month. A Cronbach’s alpha of 0.86 was found in the current study.

The GHQ-30 [22] is a screening instrument that measures symptoms of psychiatric illness. The GHQ-30 was derived from the GHQ-60 [22] and contains questions on self-rated overall health, symptoms of depression, anxiety, sleep problems, social dysfunction and somatic complaints. It generates a global measure of mental well-being. Items are rated on a 4 point Likert scale ranging from “not at all” to “more than usual”. The 30 items are recoded from 1, 2, 3, 4 to 0, 0, 1, 1 providing a total range of 0–30, where higher scores indicate more psychological distress. A Cronbach’s alpha of 0.93 was found in the current study.

Secondary outcome measures were sleep and cognitive failures described in more detail in Dalgaard et al. [17]. Sleep quality was measured with 5 items from the Danish version of the Basic Nordic Sleep Questionnaire (BNSQ) [23, 24]. The 5 items had a Cronbachs alpha of 0.67 in the current study. Cognitive failures were measured with two scales from the Cognitive Failures Questionnaire (CFQ) that addressed memory and distraction [24–27]. A Cronbach’s alpha of 0.83 was found for the memory-scale and 0.82 for the distraction-scale.

### Supplementary data

The baseline questionnaire contained all outcome measures and questions on self-reported sick-leave status (full or partial), length of sick leave, education, occupation and use of medication (as depicted in Table 1). Information on age and gender was generated from the civil registration number of the patient [28]. The psychologist registered the diagnosis at the time of clinical assessment of participants in the intervention group and control group A.

**Table 1:** Demographic and Baseline Characteristics for all groups^a b^

Characteristics	Intervention		Control group A		Control group B	
	n	%	n	%	n	%
Gender						
Female	43	74.1	40	71.4	37	75.5
Male	15	25.9	16	28.6	12	24.5
Self-reported sick leave at baseline						
Full	33	56.9	35	62.5	25	51.0
Partial	25	43.1	21	37.5	16	32.7
missing values	-	-	-	-	8	16.3
Basic education						
9th grade/less	8	13.8	5	8.9	3	6.1
10–12 years	49	84.5	50	89.3	39	79.6
missing values	1	1.7	1	1.8	7	14.3
Higher education						
Short (<3 years)	21	36.2	27	48.2	26	53.1
Medium (3–4 years)	28	48.3	24	42.9	15	30.6
Long (>4 years)	9	15.5	5	8.9	1	2.0
missing values	-	-	-	-	7	14.3
Occupation by field						
Health	5	8.6	5	8.9	6	12.2
Teaching	7	12.1	8	14.3	3	6.1
Administration	5	8.6	4	7.1	10	20.4
Day Care Worker/social	8	13.8	9	16.1	3	6.1
Leader	11	19.0	8	14.3	6	12.2
Trade/banking/it	2	3.5	8	14.3	4	8.5
Other	20	34.5	14	25.0	9	18.4
missing values	-	-	-	-	8	16.3
Diagnosis (ICD-10)						
Mild depression	16	27.6	11	19.6	-	-
Adjustment disorder/reactions to stress	42	72.4	45	80.4	-	-
Taking medication	27	44.8	25	44.6	25	51.0
Medication (by indication)						
Depression	14	24.1	11	19.6	13	26.5
Anxiety	4	6.9	5	8.9	1	2.0
Sleeping problems	6	10.3	5	8.9	8	16.3
Other	14	24.1	13	23.2	13	26.5

^a^ Mean age for the intervention group was 45 years (range 28–60 years), for control group A it was 44(range 29–63), and for control group B it was 46 (range 26–62)

^b^ Mean length of self-reported sick leave at baseline was 78 days (SD = 17 95%CI: 73–82) for the intervention group, 75 days (SD = 16, 95%CI: 71–79) for control group A, and 74 (SD = 31 95%CI: 64–84) for control group B

### Statistical analysis

Statistical analyses were conducted as close as possible to the intention-to-treat principle and thus included all data available. Statistical analyses were performed using STATA (Stata Corp. LP, College Station, TX) software package 11.2. Baseline characteristics in the groups were compared with descriptive statistics. Outcome analyses were performed with multivariate repeated measurements analysis. Due to loss to follow-up (see Figure 1) a mixed model was used in STATA to handle missing data. Model validation was performed using QQ-plots by group of residuals vs. predicted values and residual probability plots as well. A likelihood ratio test was used to test the assumption of equal standard deviations and correlations on the same subject in the different groups. Single missing responses in a scale were replaced by the mean value of the remaining items on the relevant scale for each individual. Single mean responses were only employed when more than 50% of items in a scale were available. Effect size measured by Cohen’s *d* was calculated to assess treatment effects. Calculating Cohen’s *d* is a method used to generate standardized mean differences on scales and questionnaires. Cohen’s *d* is generated by the following: d = mean difference(a) – mean difference(b)/(pooled variance of a and b). Cohen’s d is interpreted according to these guidelines: small *d* = 0.2–0.5, medium *d* = 0.5–0.8, and large *d* > 0.8 [29]. Cohen’s d was calculated so that a negative difference was equivalent to a reduction in symptoms. Spearman’s rho was carried out to test the relation between outcomes. In addition, since we experienced some loss to follow-up at 4- and 10-month follow-up, we conducted several sensitivity analyses imputing missing data on the perceived stress scale according to different scenarios: 1) last score carried forward, 2) worsening, 3) a small improvement and 4) a larger improvement over time.

### Considerations on sample size

The initial calculations showed that 300 (100 in each group) participants were needed to achieve a power of 80% and a group difference of 3 points on the PSS10 equivalent of ½ SD and 95% significance level. To account for loss to follow-up, we originally aimed at including 120 participants in each group. However, since limitations persisted in group B, the number of included participants should leave room for sufficient power in the comparisons between the intervention group and group A.

## Results

Demographic and baseline characteristics for the three groups are displayed in Table 1.

Drop-out analyses were conducted between those who responded to questionnaires at follow-up and those who did not with regard to baseline scores on outcome measures and demographic variables. There were no significant differences between those who responded to follow-up and those who did not at 4- and 10-months on outcomes at baseline or demographic variables with the exception of age, were non-responders were significantly younger at both follow-up times. This difference was further enhanced, when group B was not included. Sensitivity analyses on the PSS imputing different scenarios with regard to missing values did not change the results presented below.

Six psychologists participated in the study. Sub-analyses showed no difference in treatment effects between psychologists.

### Analyses of between group differences

Results for all outcomes are presented in the tables below (see a graphic display in Additional file 1). Mean and within-group changes from baseline to 4 months follow-up, from 4 to 10 months follow-up, as well as from baseline to 10 months follow-up are presented in Tables 2, 3 and 4 respectively.

**Table 2:** Changes over time from baseline to 4- month follow-up

	Baseline					0–4 months changes		
Variable	M	sd	95%CI	M- change	95%CI	*p*-value	d	95%CI
PSS10 (range 0–40)								
Intervention group	23.08	6.34	21.43 to 24.73	−7.31	−8.95 to −5.66	0.000	−1.15	−1.41 to −0.89
Control group A	21.76	6.28	20.07 to 23.45	−5.95	−7.71 to −4.18	0.000	−0.95	−1.23 to −0.67
Control group B	21.91	6.28	20.01 to 23.80	−4.02	−5.94 to −2.10	0.000	−0.64	−0.95 to −0.33
Effect I vs A				−1.36	−3.77 to 1.06	0.270	−0.21	−0.60 to 0 .17
Effect I vs B				−3.29	−5.81 to −0.76	0.011	−0.52	−0.92 to −0.12
Effect A vs B				−1.93	−4.54 to 0 .68	0.148	−0.31	−0.72 to 0.11
GHQ30 (0–30)								
Intervention group	19.49	7.48	17.57 to 21.41	−12.60	−14.87 to −10.32	0.000	−1.68	−1.99 to −1.38
Control group A	18.07	7.46	16.09 to 20.06	−11.48	−13.91 to −9.06	0.000	−1.54	−1.87 to −1.21
Control group B	18.60	7.45	16.35 to 20.85	−10.39	−13.05 to −7.73	0.000	−1.40	−1.75 to −1.04
Effect I vs A				−1.11	−4.44 to 2.21	0.512	−0.15	−0.59 to 0.30
Effect I vs B				−2.21	−5.71 to 1.29	0.217	−0.30	−0.76 to 0.17
Effect A vs B				−1.09	−4.70 to 2.51	0.552	−0.15	−0.63 to 0.34
BNSQ (range 5–25)								
Intervention group	17.08	4.14	16.00 to 18.17	−3.68	−4.83 to −2.53	0.000	−0.89	−1.17 to −0.61
Control group A	16.87	4.13	15.8 to 17.97	−2.86	−4.08 to −1.65	0.000	−0.69	−0.99 to −0.40
Control group B	17.20	4.12	15.96 to 18.45	−3.02	−4.36 to −1.68	0.000	−0.73	−1.06 to −0.41
Effect I vs A				−0.82	−2.49 to 0 .85	0.337	−0.20	−0.60 to 0.21
Effect I vs B				−0.66	−2.42 to 1.11	0.464	−0.16	−0.59 to 0.27
Effect A vs B				0.16	−1.65 to 1.97	0.863	0.04	−0.40 to 0.48
CFQ-Memory (0–28)								
Intervention group	10.44	5.01	9.14 to 11.74	−2.80	−3.92 to −1.68	0.000	−0.56	−0.78 to 0.33
Control group A	10.42	4.96	9.09 to 11.74	−1.55	−2.76 to −0.35	0.011	−0.31	−0.56 to −0.07
Control group B	10.59	4.93	9.1 to 12.08	−0.54	−1.87 to 0.79	0.425	−0.11	−0.38 to 0.16
Effect I vs A				−1.25	−2.89 to 0.40	0.138	−0.25	−0.58 to 0.08
Effect I vs B				−2.26	−4.00 to −0.52	0.011	−0.45	−0.80 to −0.10
Effect A vs B				−1.01	−2.81 to 0.78	0.268	−0.20	−0.57 to 0.16
CFQ-Distractions (0–36)								
Intervention group	19.68	6.06	18.11 to 21.26	−3.83	−5.26 to −2.41	0.000	−0.63	−0.87 to −0.40
Control group A	21.22	6.01	19.62 to 22.83	−3.93	−5.44 -2.42	0.000	−0.65	−0.91 to −0.40
Control group B	20.26	5.98	18.45 to 22.07	−2.95	−4.60 to −1.29	0.000	−0.49	−0.77 to −0.22
Effect I vs A				0.10	−1.98 to 2.18	0.926	0.02	−0.33 to 0.36
Effect I vs B				−0.89	−3.07 to 1.30	0.427	−0.15	−0.51 to 0.22
Effect A vs B				−0.98	−3.23 to 1.26	0.390	−0.16	−0.54 to 0.21

Effect sizes are depicted using Cohen’s d (standardized mean difference). *PSS* perceived stress scale, *GHQ* general health questionnaire, *BNSQ* basic Nordic sleep questionnaire, *CFQ* cognitive failures questionnaire, *SD* standard deviation, *CI* confidence interval, *M* mean

**Table 3:** Changes over time from 4- to 10-month follow-up

	4 months		4–10 months changes				
Variable	M	95%CI	M-change	95%CI	*p*-value	d	d 95%CI
PSS10 (range 0–40)							
Intervention group	15.77	14.1 to 17.45	−1.54	−3.24 to 0.16	0.075	−0.24	−0.51 to 0.02
Control group A	15.81	14.05 to 17.58	−1.43	−3.23 to 0.37	0.120	−0.23	−0.51 to 0.06
Control group B	17.89	15.98 to 19.80	−1.29	−3.25 to 0.67	0.198	−0.21	−0.52 to 0.11
Effect I vs A			−0.11	−2.59 to 2.36	0.928	−0.02	−0.41 to 0.37
Effect I vs B			−0.25	−2.85 to 2.34	0.849	−0.04	−0.45 to 0.37
Effect A vs B			−0.14	−2.8 to 2.52	0.919	−0.02	−0.44 to 0.40
GHQ30 (0–30)							
Intervention group	6.89	4.91 to 8.88	−1.43	−3.79 to 0.93	0.234	- 0.19	−0.51 to 0.12
Control group A	6.59	4.48 to 8.71	−1.10	−3.61 to 1.40	0.389	−0.15	−0.48 to 0.19
Control group B	8.21	5.94 to 10.49	−1.23	−3.94 to 1.48	0.374	−0.17	−0.53 to 0.20
Effect I vs A			−0.33	−3.77 to 3.11	0.850	−0.04	−0.51 to 0.42
Effect I vs B			−0.20	−3.8 to 3.39	0.912	−0.03	−0.51 to 0.45
Effect A vs B			0.13	−3.56 to 3.82	0.945	0 .02	−0.48 to 0.51
BNSQ (range 5–25)							
Intervention group	13.40	12.30 to 14.50	−1.10	−2.28 to 0.08	0.068	−0.27	−0.55 to 0.02
Control group A	14.01	12.85 to 15.17	−1.37	−2.62 to −0.13	0.031	−0.33	−0.63 to −0.03
Control group B	14.18	12.91 to 15.45	−0.92	−2.29 to 0.45	0.190	−0.22	−0.56 to 0.11
Effect I vs A			0.27	−1.44 to 1.99	0.754	0.07	−0.35 to 0 .48
Effect I vs B			−0.18	−1.99 to 1.62	0.843	−0.04	−0.48 to 0.39
Effect A vs B			−0.46	−2.31 to 1.4	0.629	0.11	−0.56 to 0.34
CFQ- Memory (0–28)							
Intervention group	7.64	6.32 to 8.96	−0.84	−2.0 to 0.32	0.158	−0.17	−0.4 to 0.06
Control group A	8.86	7.48 to 10.24	−0.88	−2.12 to 0.35	0.159	−0.18	−0.43 to 0.07
Control group B	10.05	8.54 to 11.56	−0.07	−1.43 to 1.29	0.917	−0.01	−0.29 to 0.26
Effect I vs A			0.05	−1.64 to 1.74	0.956	0.01	−0.33 to 0.35
Effect I vs B			−0.76	−2.55 to 1.02	0.402	−0.15	−0.51 to 0.21
Effect A vs B			−0.81	−2.65 to 1.02	0.386	−0.16	−0.54 to 0.21
CFQ-Distractions (0–36)							
Intervention group	15.85	14.24 to 17.46	−1.47	−2.94 to 0.004	0.051	−0.24	−0.48 to 0.001
Control group A	17.29	15.62 to 18.97	−1.28	−2.83 to 0.27	0.106	−0.21	−0.47 to 0.05
Control group B	17.31	15.5 to 19.13	−1.03	−2.72 to 0.66	0.234	−0.17	−0.45 to 0.11
Effect I vs A			−0.19	−2.32 to 1.95	0.864	−0.03	−0.38 to 0.32
Effect I vs B			−0.44	−2.68 to 1.80	0.700	−0.07	−0.45 to 0.30
Effect A vs B			−0.25	−2.55 to 2.04	0.829	−0.04	−0.42 to 0.34

Effect sizes are depicted using Cohen’s d (standardized mean difference). *PSS* perceived stress scale, *GHQ* general health questionnaire, *BNSQ* basic Nordic sleep questionnaire, *CFQ* cognitive failures questionnaire, *SD* standard deviation, *CI* confidence interval, *M* mean

**Table 4:** Changes over time from baseline to 10- month follow-up

Variable	Baseline				0–10 months changes		
	M	95%CI	M- change	95%CI	*p*-value	d-	95%Ci
Outcome							
PSS10 (0–40)							
Intervention group	23.08	21.43 to 24.73	−8.85	−10.53 to −7.16	0.000	−1.39	−1.66 to −1.13
Control group A	21.76	20.07 to 23.45	−7.38	−9.14 to −5.61	0.000	−1.16	−1.46 to −0.89
Control group B	21.91	20.01 to 23.80	−5.31	−7.24 to −3.37	0.000	−0.85	−1.15 to −0.54
Effect I vs A			−1.47	−3.91 to 0.97	0.238	−0.23	−0.62 to 0.15
Effect I vs B			−3.54	−6.11 to −0.97	0.007	−0.56	−0.97 to −0.15
Effect A vs B			−2.07	−4.69 to 0.55	0.122	−0.33	−0.75 to 0.09
GHQ30 (0–30)							
Intervention group	19.49	17.57 to 21.41	−14.03	−16.36 to −11.7	0.000	−1.88	−2.19 to −1.56
Control group A	18.07	16.09 to 20.06	−12.59	−15.03 to −10.14	0.000	−1.69	−2.02 to −1.36
Control group B	18.60	16.35 to 20.85	−11.62	−14.30 to −8.94	0.000	−1.56	−1.92 to −1.20
Effect I vs A			−1.45	−4.83 to 1.93	0.402	−0.19	−0.65 to 0.26
Effect I vs B			−2.41	−5.97 to 1.15	0.184	−0.32	−0.8 to 0.15
Effect A vs B			−0.96	−4.6 to 2.66	0.603	−0.13	−0.62 to 0.36
BNSQ (5–25)							
Intervention group	17.08	16 to 18.17	−4.78	−5.96 to −3.60	0.000	−1.15	−1.44 to −0.87
Control group A	16.87	15.8 to 17.97	−4.23	−5.45 to −3.02	0.000	−1.03	−1.32 to −0.73
Control group B	17.20	15.96 to 18.45	−3.94	−5.28 to −2.6	0.000	−0.96	−1.28 to −0.63
Effect I vs A			−0.54	−2.24 to 1.15	0.528	−0.13	−0.54 to 0.28
Effect I vs B			−0.84	−2.63 to 0.94	0.355	−0.20	−0.64 to 0.23
Effect A vs B			−0.30	−2.11 to 1.51	0.747	0.07	−0.51 to 0.37
CFQ-Memory (range 0–28)							
Intervention group	10.44	9.14 to 11.74	−3.64	−4.79 to −2.48	0.000	−0.73	−0.96 to −0.50
Control group A	10.42	9.09 to 11.74	−2.44	−3.64 to −1.24	0.000	−0.49	−0.73 to - 0.25
Control group B	10.59	9.10 to 12.08	−0.61	−1.95 to 0.72	0.369	−0.12	−0.40 to 0.15
Effect I vs A			−1.20	−2.87 to 0.47	0.159	−0.24	−0.57 to 0.09
Effect I vs B			−3.02	−4.79 to - 1.26	0.001	−0.61	−0.96 to −0.25
Effect A vs B			−1.83	−3.62 to −0.03	0.047	−0.37	−0.73 to −0.01
CFQ- Distractions (0–36)							
Intervention group	19.68	18.11 to 21.26	−5.30	−6.75 to −3.84	0.000	−0.87	−1.11 to −0.63
Control group A	21.22	19.62 to 22.83	−5.21	−6.72 to −3.70	0.000	−0.87	−1.12 to −0.61
Control group B	20.26	18.45 to 22.07	−3.97	−5.64 to −2.31	0.000	−0.66	−0.94 to −0.39
Effect I vs A			−0.09	−2.19 to 2.01	0.934	−0.01	−0.36 to - 0.33
Effect I vs B			−1.33	−3.54 to 0.88	0.240	−0.22	−0.59 to - 0.15
Effect A vs B			−1.24	−3.49 to 1.01	0.281	−0.21	−0.58 to - 0.17

Effect sizes are depicted using Cohen’s d (standardized mean difference). *PSS* perceived stress scale, *GHQ* general health questionnaire, *BNSQ* basic Nordic sleep questionnaire, *CFQ* cognitive failures questionnaire, *SD* standard deviation, *CI* confidence interval, *M* mean

There were no significant outcome differences between the intervention group and control group A (receiving assessment alone) at any time point. The intervention group consistently exhibited larger mean change values from baseline to 4 months follow-up than the two other groups on all outcome measures except for CFQ-distraction, where the mean-change value in control group A was a little higher. However, effect sizes were small in all cases. When compared to control group B, the intervention group exhibited significantly larger reductions in levels of perceived stress and memory complaints at both 4- and 10-month follow-up, yielding moderate effect sizes (Cohen’s d). With the exception of sleep at 4-month follow-up, control group A displayed larger mean changes for all outcomes and time points when compared to control group B, but between group differences were only significant with regard to memory at 10-month follow-up, where a small to moderate effect size was found.

Within-group results are also shown in Tables 2, 3 and 4. All groups significantly improved on all psychological complaints from baseline to 4-month follow-up except with regard to CFQ-memory in control group B. Effect sizes at 4-months follow-up were moderate to large for all outcomes in all groups with the exception of memory, where effect sizes were small in control groups A and B. Similar improvements were not observed between 4- and 10-month follow-up.

All measures were highly and significantly correlated (Spearman’s correlations not shown), but in particular, PSS and GHQ and PSS and CFQ- Distraction and – Memory, respectively, yielded strong correlations.

## Discussion

The intervention was not more effective in reducing perceived stress and psychological complaints when compared to control group A that received clinical assessment. A few outcome differences were found between the intervention group and control group B, but they should most likely not be attributed to the intervention.

One reason behind the lack of intervention effect, when the intervention group was compared to group A, could be a high degree of natural recovery. The majority of participants were diagnosed with adjustment disorder, a condition where symptoms normally improve within a period of 6 months. Consequently, all groups improved significantly over time, a tendency also seen in several other studies [15, 16]. Since all patients in this study were on sick leave when included, they could rest and avoid excessive work demands; this should enhance a natural recovery process. Although stress levels improved during 10 months follow-up, levels may not have reached the levels of the general population. Scores on the PSS10 at 10-month follow-up (disregarding control group B) remained almost 4 points higher than the mean score of a comparable non-stress control group employed in another study from our department [30]. The difference was equivalent to a moderate effect size (Cohen’s d = 0.6). Elevated symptom levels at 10-month follow-up have also been observed in other studies [16], but it remains unclear if this is a result of work-related stress, or if individuals suffering from work-related stress had elevated symptoms before the stress episode.

The intervention tested in the current study was previously evaluated in a two-armed RCT study, where patients were referred through their general practitioner. In that study a significant treatment effect was found on both the PSS and the GHQ corresponding to moderate effect sizes (D.J. Glasscock, personal communication September 1, 2016). The sample in the current study had on average been on sick leave for more than 2 months when included in the study. In contrast the study sample in the previous study had only been on sick leave for on average about 40 days, when included. In accordance with the notion of natural recovery above, the difference in length of sick leave at baseline may explain the different results. Thus employing CBT after about 2 month of sick leave among participants with adjustment disorders may be too late to alter the speed of symptom recovery. On the other hand, other results from the present study (not reported here) concerning return to work suggest that patients receiving the intervention were able to terminate sick-leave about 4 weeks earlier than control group A (Dalgaard et al. [Return to work after work-related stress: A randomized controlled trial of a work-focused cognitive behavioural intervention, *in press*]). Again this may indicate that the timing of the intervention is important. An earlier intervention might support faster recovery while delayed intervention is not able to demonstrate any improvement over and above that which occurs naturally. On the other hand, once sufficient recovery has taken place, patients may be more able to benefit from support directed at the return to work process.

Nonetheless, our current results are in line with a few other comparable RCT studies, where no treatment effects of various CBT-inspired approaches were found on psychological complaints [15, 16]. CBT may be more effective in reducing symptoms in patients with more serious psychiatric conditions like depression or anxiety, and perhaps in less chronic conditions, where work-related stress has not yet necessitated sick leave. A few other studies have found CBT to be superior in treating work-related stress complaints in sick-listed patients [18, 19] using a wait-list controlled design. However, these results should perhaps be interpreted with caution since a recent study indicates that wait-list controlled trials could induce a “nocebo” effect in control groups due to expectation of later recovery [31].

The majority of participants in the current study were female workers from 30 to 60 years of age employed in the Danish public sector. We cannot say if individual factors such as for example menopause have influenced outcomes. However, the successful randomization procedure has ensured that any such influences are equally represented in the two main groups and can thus not have affected between group comparisons. However, it is unclear to what extent our results may be generalized to male workers, workers outside of the public domain or workers in countries where sick-leave policies differ substantially from Danish regulations. It may be noted that our sample very closely resembles patients routinely referred to the department because of work-related stress by general practioners.

While great effort was made via clinical assessment to ensure a well-defined sample, the patients were still heterogeneous in terms of symptoms and stressors. Some patients displayed depressive mood and exhaustion, while others experienced heightened anxiety, sleep problems or cognitive difficulties. This heterogeneity might also pose a barrier for detecting a treatment effect and it is highly likely that distinct subgroups within the population exist. This problem of heterogeneity is often present in comparable studies to a greater extent because of a lack of clinical assessment. Indeed the term work stress, which is often used, is somewhat vague and probably covers a wide range of conditions and work situations. For this reason researchers need to be more specific when defining concrete samples. Subgroups may well exist not only in terms of presenting symptoms and chronicity of the condition, but also with regard to the types of stressors that are assumed to underlie the condition, e.g. work overload, bullying, role conflict. The present and other interventions may have a beneficial effect in some subgroups but not others. However, our sample size is insufficient to conduct subgroup analyses.

The results from this and several other RCT’s with similar samples are somewhat disappointing with regard to the treatment effects of CBT. As we have noted, disappointing results may be due to the timing of the intervention with regard to the recovery process, a lack of treatment efficiency or limitations such as small sample sizes and lack of genuine no-treatment control groups. We also feel that the heterogeneity of samples labelled with the term ‘work stress’ may be a barrier. As noted by de Vente et al. [16], subgroups may matter with regard to treatment effectiveness. The identification of more homogenous subsamples relating to symptoms or different types of stressful conditions might create conditions better suited to detecting treatment effects. This would also make comparisons between studies easier. For example, patients whose stress condition follows a period of workplace bullying might have different treatment needs to those whose symptoms arise following a period of extreme workload. Another important distinction concerns the duration of a stress-related condition. Creating study samples containing both patients with recently developed symptoms and patients with more chronic conditions may mask true treatment effects that apply to only one of these groups. While the baseline clinical assessment in the present study was intended to reduce such heterogeneity, more could be done to identify relevant subgroups. This would of course require larger samples. Future studies are needed to address these areas.

### Strength and limitations

The main strength of the study is the randomized controlled design. A further strength is the thorough baseline examination which increases confidence in the likelihood that patients in the intervention group and control group A were actually on sick leave due to work-related stress. We also consider it to be a strength that our intervention had a dual focus involving both the individual and working conditions.

Several limitations should also be addressed. First, the lack of clinical assessment in group B probably caused selection bias in this group. Thus, control group B likely contained patients, whose condition was insufficiently related to work stress and/or patients with other psychiatric illness (i.e., major depressive episode). This seems probable given the numbers excluded for these reasons during clinical assessment in the two other groups. Consequently, it is important to underline that clinical assessment at baseline is pivotal to ensure that those included in similar RCT’s meet inclusion criteria.

Second, selection bias may also have taken place during the screening procedure (see Figure 1), since many did not return the screening questionnaire. We do not know whether non-responders were more or less stressed than those who participated. The only available data showed that non-responders were significantly younger than responders but did not differ with regard to gender.

Third, the use of professional help outside of the study may have impacted chances of detecting a treatment effect. Data at 4-month follow-up revealed that 36 participants in control group A and 28 in control group B had received psychological treatment outside of the study during the previous 6 months. Furthermore, 21 participants in the intervention group also reported having seen a psychologist outside of the study. We do not know to what extent external help happened before or after inclusion into the study. However, the use of professional assistance was three times as large in the two control groups as in the intervention group from 4 to 10 months follow-up, possibly reflecting a greater need in the control groups. During the study period, there has been extensive focus on stress-related sick leave in the media. Organizations have increasingly utilized private health insurance policies that provide easier access to psychological assistance. As a consequence, many psychologists in the private sector may have gained more experience in addressing stress-related complaints. For ethical reasons it was not feasible to prohibit patients from seeking care elsewhere. We are not aware of any similar trials (except from Blonk et al. [15]) that have been able to employ a no treatment control group unless a wait list controlled design was used. Therefore the control groups might best be perceived as care as usual groups.

A fourth, limitation concerns loss to follow-up, especially in the two control groups. However, as mentioned earlier, sensitivity analyses accounting for various scenarios concerning stress levels on the PSS scale did not alter our results. We therefore consider it less likely that drop-out caused systematic bias.

Fifthly, the intervention offered a workplace intervention together with the individual program, which was mentioned as a strength. However, a direct workplace intervention was only possible in 6 cases. Some patients resisted bringing a psychologist into the workplace, perhaps because they felt this might have a stigmatizing effect. However, the work-focus was maintained in the individual sessions.

Finally, the small sample size, which was partly due to subjects being excluded following clinical assessment, may have limited the power to detect a treatment effect. These limitations may increase the risk of wrongly rejecting work-focused CBT as a viable treatment option for this patient group.

## Conclusion

Six sessions of work-focused CBT and the offer of a short workplace intervention was not more effective than control condition A, which received a clinical assessment at baseline. Some outcome differences were observed between the intervention group and control group B, but these cannot be attributed to the intervention. Psychological complaints improved over time in all three groups.

## Additional file

Additional file 1: Mean changes on psychological outcome measures from baseline through 4 and 10 month follow-up. (PDF 381 kb)

## Abbreviations

BNSQ: Basic Nordic sleep questionnaire
CBT: Cognitive behavioral therapy
CFQ: Cognitive failures questionnaire
CI: Confidence interval
GHQ: General health questionnaire
M: Mean
OP: Occupational physician
PSS: Perceived stress scale
RCT: Randomized controlled trials
SD: Standard deviation

## References

[1] Van der Klink JJ, Blonk RWB, Schene AH (2001). The benefits of interventions for work-related stress. Am J Public Health.

[2] Arends I, Bruinvels DJ, Rebergen DS (2012). Interventions to facilitate return to work in adults with adjustment disorders. Cochrane Database Syst Rev.

[3] Kompier M (2006). New systems of work organization and workers’ health. Scand J Work Environ Health.

[4] Landsbergis P (2003). The changing organization of work and the safety and health of working people: a commentary. J Occup Environ Med.

[5] Bhagat RS (2012). Work Stress and Coping in the Era of Globalization.

[6] Nielsen MB, Bültmann U, Madsel IEH (2012). Health, work, and personal-related predictors of time to return to work among employees with mental health problems. Disabil Rehabil.

[7] The ICD-10 Classification of Mental and Behavioural Disorders (1992). Clinical Descriptions and Diagnostic guidelines.

[8] Lazarus RS, Folkman S (1984). Stress, appraisal, and coping.

[9] Cox T, Ferguson E, Cooper C, Payne L (1991). Individual difference, stress and coping. Personality and Stress: Individual Differences in the stress process.

[10] Lazarus R. Stress and Emotion: A New Synthesis. New York: Springer Publishing Company; 1999.

[11] McEwen B (2005). Stressed or stressed out: What is the difference?. J Psychiatry Neurosci.

[12] Richardson KM, Rothstein HR (2008). Effects of occupational stress management intervention programs: a meta-analysis. J Occup Health Psychol.

[13] Bhui KS, Dinos S, Stansfeld SA (2012). A synthesis of the evidence for managing stress at work: a review of the reviews reporting on anxiety, depression, and absenteeism. J Environ Public Health.

[14] van der Klink JJ, Blonk RW, Schene AH (2003). Reducing long term sickness absence by an activating intervention in adjustment disorders: a cluster randomised controlled design. Occup and Environ Medicine.

[15] Blonk RWB, Brenninkmeijer V, Lagerfeld SE (2006). Return to work: A comparison of two cognitive behavioural interventions in cases of work-related psychological complaints among the self-employed. Work Stress.

[16] de Vente W, Kamphuis JH, Emmelkamp PM (2008). Individual and group cognitive-behavioral treatment for work-related stress complaints and sickness absence: a randomized controlled trial. J Occup Health Psychol.

[17] Dalgaard L, Eskildsen A, Carstensen O (2014). Changes in self-reported sleep and cognitive failures: a randomized controlled trial of a stress management intervention. Scand J Work Environ Health.

[18] Willert MV, Thulstrup AM, Hertz J (2009). Changes in stress and coping from a randomized controlled trial of a three-month stress management intervention. Scand J Work Environ Health.

[19] Willert M, Thulstrup AM, Hertz J (2010). Sleep and Cognitive Failures Improved by a Three-Month Stress Management Intervention. Int J Stress Manag.

[20] Netterstrøm B, Friebel L, Ladegaard Y (2013). Effects of a multidisciplinary stress treatment programme on patient return to work rate and symptom reduction: results from a randomised, wait-list controlled trial. Psychother Psychosom.

[21] Cohen S, Williamson G, Spacapam S, Oskamp S (1988). Perceived stress in a probability sample of the United States. The social psychology of health: Claremont Symposium on applied social psychology.

[22] Goldberg DP, Williams P (1988). A user’s guide to the General Health Questionnaire.

[23] Partinen M, Gislason T (1995). Basic Nordic Sleep Questionnaire (BNSQ): a quantitated measure of subjective sleep complaints. J Sleep Res.

[24] Broadbent DE, Cooper PF, FitzGerald P (1982). The Cognitive Failures Questionnaire (CFQ) and its correlates. Br J Clin Psychol.

[25] Wallace JC, Kass SJ, Stanny CJ (2002). The cognitive failures questionnaire revisited: dimensions and correlates. J Gen Psychol.

[26] Wallace JC (2004). Confirmatory factor analysis of the cognitive failures questionnaire: evidence for dimensionality and construct validity. Pers Individ Dif.

[27] Wilhelm O, Witthöft M, Schipolowski S (2010). Self-reported Cognitive Failures Competing measurement Models and SelfReport Correlates. J Ind Diff.

[28] Schmidt M, Pedersen L, Sørensen HT (2014). The Danish Civil Registration system as a tool in epidemiology. Eur J Epidemiol.

[29] Cohen J (1988). Statistical power analysis for the behavioral sciences.

[30] Eskildsen A, Andersen LP, Pedersen AD (2015). Work-related stress is associated with impaired neuropsychological test performance: a clinical cross-sectional study. Stress.

[31] Furukawa TA, Noma H, Caldwell DM (2014). Waiting list may be a nocebo condition in psychotherapy trials: a contribution from network meta-analysis. Acta Psychiatr Scand.

