# Supplementary material for: Work-focused cognitive behavioral intervention for psychological complaints in patients on sick leave due to work-related stress: Results from a randomized controlled trial
Source: J Negat Results Biomed. 2017 Aug 22;16:13. doi: 10.1186/s12952-017-0078-z (PMC5567478; doi:10.1186/s12952-017-0078-z)
Supplement: Additional file 1: — Mean changes on psychological outcome measures from baseline through 4 and 10 month follow-up. (PDF 381 kb) [file 12952_2017_78_MOESM1_ESM.pdf]

Additional file 1

Mean changes on psychological outcome measures from baseline through 4- and 10-month follow-up

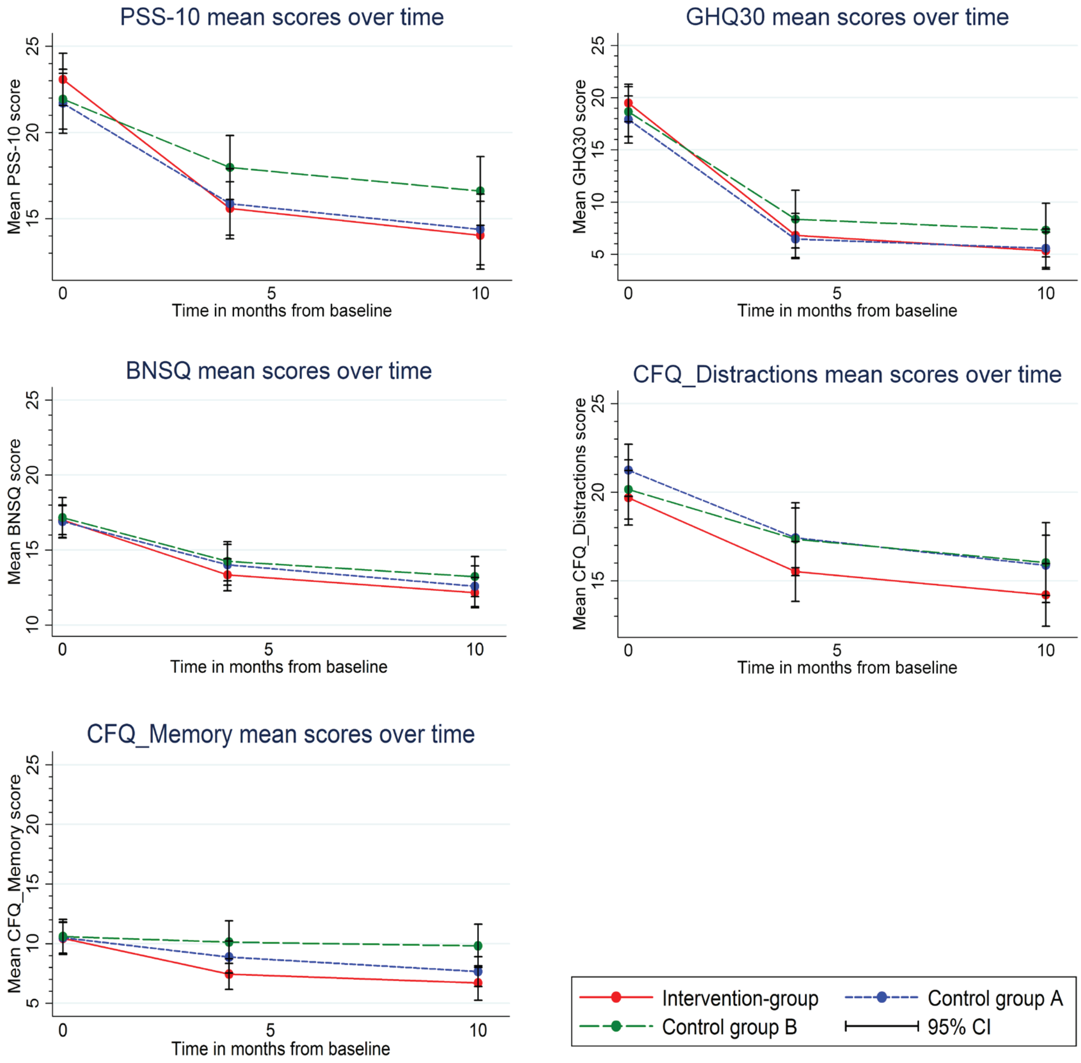

PSS=Perceived Stress Scale, GHQ=General Health Questionnaire, BNSQ=Basic Nordic Sleep Questionnaire, CFQ=Cognitive Failures Questionnaire, CI=Confidence Interval.
